# The Effectiveness of a Race-Based Stress Reduction Intervention on Improving Stress-Related Symptoms and Inflammation in African American Women at Risk for Cardiometabolic Disease: Protocol for Recruitment and Intervention for a Randomized Controlled Trial

**DOI:** 10.2196/65649

**Published:** 2025-04-18

**Authors:** Karen L Saban, Cara Joyce, Alexandria Nyembwe, Linda Janusek, Dina Tell, Paula de la Pena, Darnell Motley, Lamise Shawahin, Laura Prescott, Stephanie Potts-Thompson, Jacquelyn Y Taylor

**Affiliations:** 1 Center for Translational Research and Education Loyola University Chicago Marcella Niehoff School of Nursing Maywood, IL United States; 2 Loyola University Chicago Parkinson School of Health Sciences and Public Health Maywood, IL United States; 3 University of California, Irvine Irvine, CA United States; 4 University of Chicago Department of Medicine Chicago, IL United States; 5 Governors State University College of Education and Human Development University Park, IL United States; 6 Center for Research on People of Color Columbia University School of Nursing New York, NY United States

**Keywords:** cardiometabolic disease, stress, Resilience, Stress, and Ethnicity, RiSE intervention, health of minoritized groups

## Abstract

**Background:**

In recent years, the prevalence of cardiometabolic disease (CMD) in African American women has risen; the risk also increases with age, in comparison to men. Evidence demonstrates that stressful life events, including experiences of racism and perceived discrimination, contribute substantially to inflammatory diseases, such as CMD. Despite this evidence, few evidence-based interventions are available to assist individuals from minoritized communities in coping with the chronic stress related to their racial or ethnic identity.

**Objective:**

Our proposed randomized controlled trial will test a novel, race-based intervention tailored to African American women, called Resilience, Stress, and Ethnicity (RiSE).

**Methods:**

In this randomized controlled trial, we will randomize participants 1:1 to the 8-week, group-based RiSE program (intervention) or a health education program (active control group). Both programs will consist of synchronous classes on Zoom and will be led by experts. The primary end point will be stress at 6 months after the intervention, and the efficacy of RiSE will be evaluated for improving stress-related symptoms (current perceived stress, depressive symptoms, fatigue, and sleep disturbance), improving coping strategies, and reducing inflammatory burden in African American women at risk for CMD. Validated survey measures and inflammatory biomarkers will be assessed at baseline, midintervention, intervention completion, and 6 months after the intervention, and differences over time by intervention will be evaluated using mixed effects models.

**Results:**

This study was funded by the National Institute on Aging on March 30, 2023, with recruitment and enrollment beginning in October 2023. The study is underway, with 120 participants enrolled as of March 2025.

**Conclusions:**

This study will be one of the first to examine a race-based stress reduction intervention in African American women and has the potential to improve the health of minoritized groups faced with chronic stress associated with experiencing racism and discrimination. We anticipate that RiSE will reduce stress-related symptoms, enhance adaptive coping, and reduce inflammation.

**Trial Registration:**

ClinicalTrials.gov NCT05902741; https://www.clinicaltrials.gov/study/NCT05902741

## Introduction

### Cardiometabolic Disease Among African American Women

Cardiometabolic disease (CMD) is a complex pathophysiological syndrome characterized by progressive abdominal adiposity, dyslipidemia, hypertension, and insulin resistance [1,2]. It is well established that CMD heightens the risk for cardiovascular disease (CVD) [3]. Individuals with CMD have a 2-fold risk of mortality from coronary artery disease and a twice greater risk of stroke [4]. In recent years, the prevalence of CMD in women has risen drastically in comparison to men [3], and the risk increases with age [5]. Studies demonstrate that females experience more sex-specific risk factors that increase adiposity [6], resulting in greater risk for CMD [7,8]. Furthermore, CMD is disturbingly prevalent in African American women; for example, 55% of non-Hispanic Black women in the United States are obese [9], 43% have hypertension [10], and 13% have been diagnosed with diabetes mellitus [11]. While CVD mortality has declined in the United States, these same reductions have not been seen in African American women who continue to experience a disproportionate CVD burden compared to their White counterparts, even after controlling for traditional risk factors, such as smoking and weight [12].

### Psychosocial Stressors, Psychobiological Response, and CMD

Numerous studies link higher levels of psychosocial stress to both CMD and CVD [13-17]. Recent evidence demonstrates that stressful life events, including experiencing racism and perceived discrimination, play a substantial role in contributing to inflammatory diseases, such as CMD [18,19]. African Americans who reported a history of experiencing racism had a significantly greater incidence of carotid artery plaque [20] and hypertension [21] than those who did not. African American women experience greater social stress from exposure to both racism and sexism [22,23]. Furthermore, low socioeconomic status may contribute to chronic stress in African American women, thereby increasing the risk of CMD [24]. Adaptive coping (seeking social support, problem-solving, and resistance to racism) may reduce stress-related symptoms (current perceived stress, depressive symptoms, fatigue, and sleep disturbance) and inflammatory burden [25-27] and moderate the impact of racism on well-being and health [28-30]. Furthermore, evidence demonstrates that there are sex differences in the types of coping strategies used, particularly in response to racial discrimination; for example, while men tend to use suppression and distraction, women are more prone to rumination [31]. Importantly, individuals can learn adaptive coping strategies that result in decreased stress-related symptoms [32], inflammatory burden (ie, proinflammatory cytokines) [33], and CVD [34].

### The Need for a Novel Intervention to Ameliorate Psychobiological Stress and Empower African American Women

Despite evidence that chronic stress associated with experiencing racism, discrimination, and sexism increases the inflammatory response, few evidence-based interventions are available to assist individuals from minoritized communities in coping with the chronic stress related to their racial or ethnic identity. Interventions to assist individuals in managing chronic stress, such as mindfulness-based stress reduction (MBSR) and cognitive behavioral therapy (CBT) do not address trauma related to experiencing racism and discrimination [35]. Furthermore, MBSR and CBT do not specifically address race-based stressors. To date, only 3 published interventions address coping with discrimination; 2 interventions focus on coping related to discrimination experienced by individuals positive for HIV [36], and the other addresses racial socialization within Black families [37]. However, none of these interventions specifically address racism and discrimination experienced by African American women at risk for CMD. As research related to discrimination grows, there is a significant need for evidence-based interventions to address the psychobiological and health consequences of discrimination at the individual level [38].

Our proposed randomized controlled trial (RCT) will test a race-based intervention tailored to African American women, called Resilience, Stress, and Ethnicity (RiSE) [35] (Motley D, unpublished data, November 2017). In this group-based, 8-week intervention, cognitive behavioral and stress reduction strategies will be integrated [39] focusing on the biopsychosocial effect of racism [40,41], racial identity development [42-44], and empowerment [45]. Table 1 presents the schedule of enrollment, interventions, and assessments for the study.

**Table 1:** Study schedule of enrollment, interventions, and assessments.

	Enrollment	Baseline (T0)	Midintervention (T1)	Intervention completion (T2)	6 months after intervention completion (T3)
Eligibility screen	✓				
Informed consent		✓			
First DNA sample		✓			
Allocation		✓			
Health measures		✓	✓	✓	✓
Audio computer-assisted self-interview		✓	✓	✓	✓
Second DNA sample					✓
Final assessment					✓

Our previous pilot study found that RiSE improved the ability of African American women to cope with racism and increased the use of adaptive coping strategies through emotional validation and cognitive restructuring of the response to racism and discrimination [35]. However, no research has examined RiSE as an intervention for the reduction of stress-related symptoms and inflammation in African American women at risk for CMD. The psychobiological impact of RiSE and its potential to reduce health risks may improve the health of many minoritized groups who endure chronic discrimination and racism [35]. Importantly, our study addresses the impact of racism on the individual that has not previously been addressed by other interventions.

On the basis of our preliminary data as well as evidence from other stress reduction interventions [46,47], we propose that RiSE will improve stress-related symptoms by providing access to coping strategies that may decrease inflammation (as indicated by biomarkers such as C-reactive protein [CRP], interleukin-6 [IL-6], tumor necrosis factor–alpha [TNF-α], interleukin-1beta [IL-1β], and interferon-gamma [IFN-γ]). Our pilot data indicate that RiSE attenuates maladaptive coping [48]. Adaptive coping, such as seeking social support and active coping, has been found to lessen the harmful effects of perceived discrimination and racism on the health of minoritized groups [49,50]. Furthermore, adaptive coping is related to well-being and lower levels of inflammation [51].

### Conceptual Model

**Figure 1:**
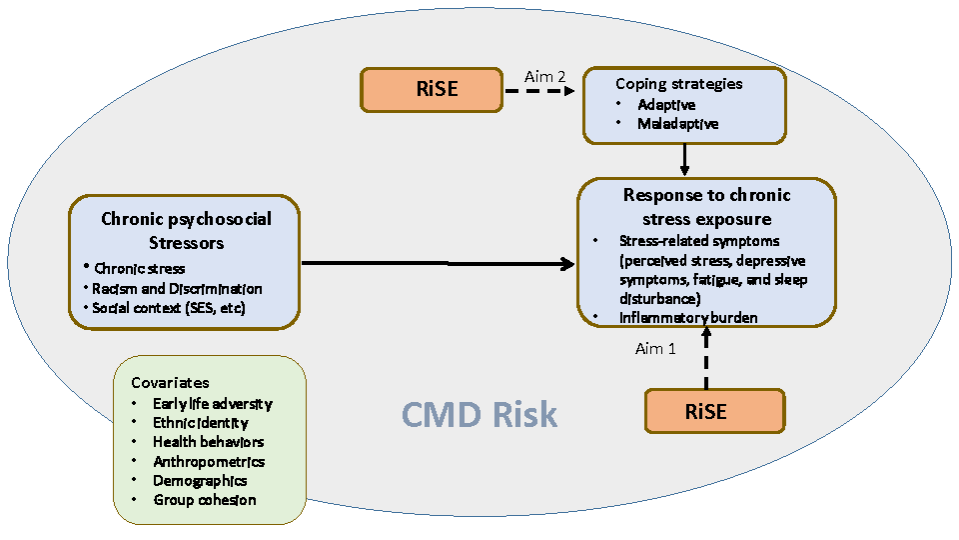
Conceptual model. CMD: cardiometabolic disease; RiSE: Resilience, Stress, and Ethnicity; SES: socioeconomic status.

Our conceptual model (Figure 1) is based on allostatic load theory [52], which posits that chronic stressors produce wear and tear on the body through prolonged hyperactivity or hypoactivity of adaptive physiological systems.

There are 3 types of responses to stressors: frequent activation of the stress response (eg, responding to the frequency and severity of a stressor), failed response (eg, chronic response where the body is unable to stop reacting to the stressor), and inadequate response (eg, the body has little to no response to the stressor) [52]. Our model proposes that psychosocial stressors, such as discrimination, racism, and social context (eg, socioeconomic precarity) when chronic, repeated, and overbearing, may heighten inflammation [53-55].

In this paper, we present a research protocol for the primary aim: to investigate the impact of RiSE on the response to chronic psychosocial stressors and inflammation. The subaim is to explore the impact of RiSE on adaptive coping (ie, problem- and emotion-focused coping strategies, such as positive mindset reframing and venting) versus maladaptive coping strategies) [55]. We hypothesize that women who participate in the RiSE intervention will exhibit decreases in stress-related symptoms (current perceived stress, depressive symptoms, fatigue, and sleep disturbance) as well as decreases in inflammatory burden (as indicated by biomarkers such as IL-6, TNF-α, IL-1β, IFN-γ, and CRP) compared to women in the health education program (HEP), which is the active control arm. For the subaim, we anticipate that women who participate in the RiSE intervention will report an increase in the use of adaptive coping strategies (seeking social support, problem-solving, and resistance to racism) and a decrease in the use of maladaptive coping strategies (avoidance and internalizing racism). This work is innovative because we are working to add resources to the toolbox of minoritized groups to increase resiliency in the face of racism at the interpersonal level. Although successful in some populations and types of stress-related conditions, CBT and MBSR were not specifically developed to reduce the impact of racism and discrimination [35]. RiSE has the potential to reduce the psychobiological consequences of racism and discrimination. This research study should aid in the continuing efforts of researchers to provide tools, which can be implemented as necessary, to groups considered marginalized.

## Methods

### Ethical Considerations

This study was approved by the Loyola University Chicago Health Sciences Institutional Review Board (LU214133) and the WCG Institutional Review Board (20230283). Eligible and willing participants will provide written informed consent. Participants can choose to opt out at any point during the study. All data are deidentified. Given the extent of participant involvement, we ensured fairness in the compensation process. The participants can be compensated up to US $500 in electronic Amazon gift cards. Compensation transparency is ensured. The recruitment flyers, telephone screen, and consent form all contain the mode of compensation and the amount. The study has been registered at ClinicalTrials.gov (NCT05902741).

### Design

**Figure 2:**
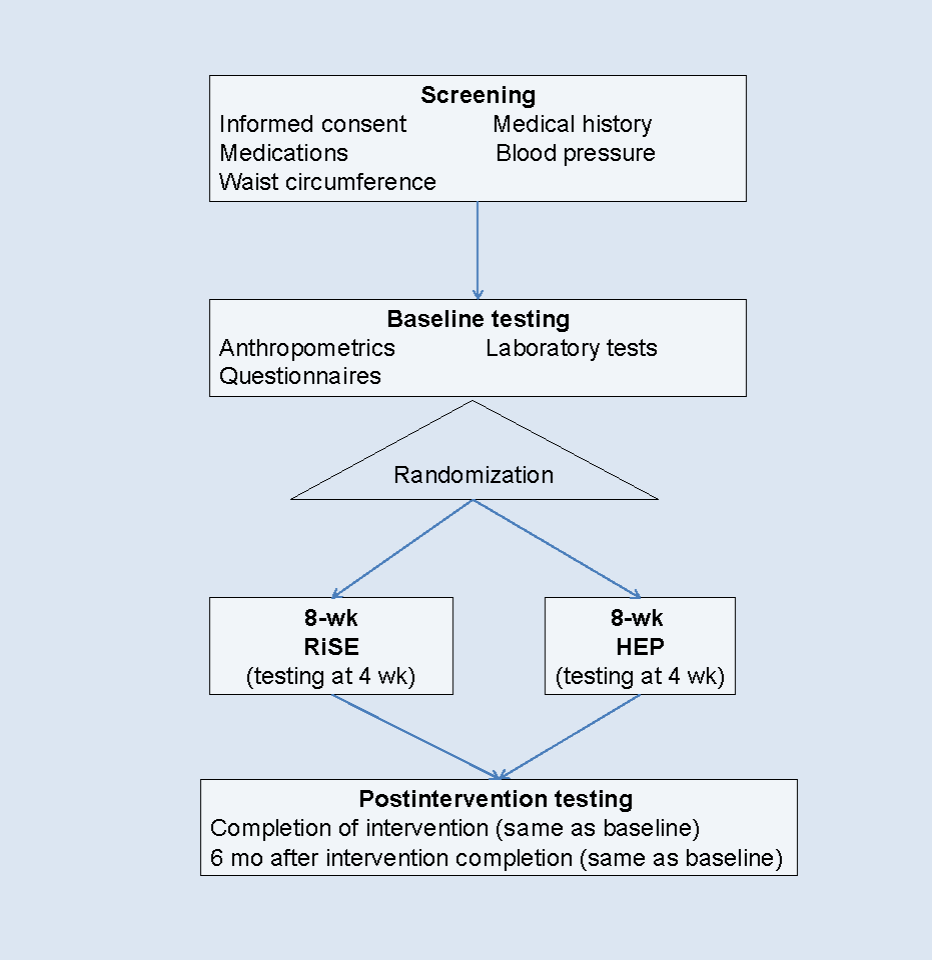
Overview of the study design. HEP: health education program; RiSE: Resilience, Stress, and Ethnicity.

In this RCT (stage II [traditional efficacy testing] of the National Institutes of Health Stage Model for Behavioral Intervention Development [56,57]), we will determine the effectiveness of the 8-week, group-based RiSE program (intervention) in improving stress-related symptoms (current perceived stress, depressive symptoms, fatigue, and sleep disturbance), reducing inflammatory burden, and improving coping strategies from baseline to after the intervention in African American women aged 50 to 75 years at risk for CMD. This age range was chosen because women develop a higher risk for CMD [58] during perimenopause [59]. We are excluding women aged >75 years to control for the effects of older age on inflammation and DNA methylation [60,61]. Outcomes from the intervention group (RiSE) will be compared to those from the active control group (HEP). Figure 2 presents the overview of the study design.

### Procedures

We will recruit 300 African American women at risk for CMD from 2 large metropolitan cities in the United States (Chicago and New York City). Both communities are ethnically and racially diverse with relatively large African American populations [62,63].

We will use several recruitment strategies. First, we will work closely with key trusted community leaders (eg, church leaders) to promote the study. This strategy is particularly effective in recruiting people from minoritized communities in clinical trials [64,65]. In addition, we will meet regularly with our community advisory board, made up of individuals from the surrounding community, for suggestions on how best to recruit potential participants. We will send recruitment letters describing the study to African American women (aged 50-75 y) who broadly meet eligibility criteria (eg, no history of myocardial infarction or ischemic stroke) from our respective hospital databases. In addition, we will recruit women through community clinics, churches, health fairs, hair salons, social media, newspaper advertisements, and word of mouth. The use of proven recruitment and retention strategies for research participants from minoritized communities [64,66,67] will also be advantageous for this study. These efforts include sharing health information about CMD, engaging community representatives through the sharing of health information, and providing cultural sensitivity training for the study team [68-70]. Potential study participants will be prescreened for eligibility over the telephone. An appointment will be scheduled for further screening for those meeting the study eligibility criteria. Textbox 1 presents detailed inclusion and exclusion criteria.

**Inclusion and exclusion criteria.**

**Inclusion criteria**

- Aged between 50 and 75 years
- Woman
- Postmenopausal stage (without menstrual periods for at least 12 consecutive months)
- Self-identify as African American
- Able to read, write, and speak English
- At least one of any of the following: waist circumference >88 cm; systolic blood pressure >130 mm Hg and diastolic blood pressure >88 mm Hg, or use of antihypertensive medications; diagnosed and receiving treated for hypercholesterolemia; history of type 2 diabetes

**Exclusion criteria**

- History of myocardial infarction or ischemic heart disease and angina pectoris, stent placement, coronary artery bypass, left ventricular hypertrophy, congestive heart failure, or ischemic stroke
- Any major immune-related disease (eg, rheumatoid arthritis or lupus)
- Use of immune-altering medications, such as glucocorticoids
- Periodontal disease, bleeding gums, and dental work in the past 72 h
- Current smoker or has smoked in the past 3 mo
- Active cancer
- Active infection
- Substance abuse
- Cognitive or psychiatric disorder that would affect the ability to participate in program classes (Brief Screen for Cognitive Impairment total score of ≥8)

Participants will be assessed for CMD risk. They will also be assessed for cognitive impairment using the 3-item Brief Screen for Cognitive Impairment [71]: a total score of ≥8, which suggests cognitive impairment, will lead to the exclusion of the participant from the study. All ineligible participants will receive a gift card (US $10) after the screening. Baseline measures will be collected after the screening.

After the baseline interview (T0), eligible participants will be randomized 1:1 to RiSE or HEP, with each wave of enrollment treated as a block. The randomization plan (PROC PLAN) generated in SAS 9.4 (SAS Institute Inc) includes the site as a stratifying variable, and we plan for each site to have at least 6 RiSE and 6 control participants per wave. Patient registration data, group assignment, and self-report survey data will be managed via a single REDCap (Research Electronic Data Capture; Vanderbilt University) server with access for both sites.

Assessments are completed face-to-face in a private room of our research building. At each of the 4 assessment points (T0: baseline, T1: midintervention; 4 wk, T2: intervention completion; 8 wk, and T3: 6 mo after intervention completion), trained interviewers will collect the following anthropometric measurements: 3 blood pressure readings, waist and hip circumference, weight, height, body fat percentage, and body water percentage. Data on psychosocial factors, health behaviors, and medical history will be collected using an audio computer-assisted self-interview system [72] and REDCap surveys. In addition, we will collect saliva samples using a passive drool kit to measure participants’ inflammatory burden (as indicated by biomarkers such as CRP, IL-6, TNF-α, IL-1β, and IFN-γ) [46,51,73-82]. At T0 and T3 (ie, baseline and final assessment; 6 mo after intervention completion, respectively), we will collect participants’ saliva samples using the Oragene DNA kit (DNA Genotek) to measure DNA methylation [68]. Study participants will receive a gift card (US $50) after the completion of each interview (T0-T3).

### Intervention

#### Overview

Both RiSE and HEP groups will meet weekly on Zoom (Zoom Communications, Inc) for 8 consecutive weeks for approximately 2 hours per session. In addition, 2 sessions will occur 1 month and 2 months after the completion of the interventions. These booster sessions will include a brief review of information covered during the programs and will allow the participants the opportunity to discuss and ask questions about how they applied what they learned in their lives. For every class attended, participants will receive a US $30 gift card. Participants will be sent an SMS text message or email reminder before each class. The RiSE and HEP classes will meet synchronously over Zoom and will be led by experts in the area. The study staff will review how to access Zoom with research participants at the baseline data collection appointment and will answer any questions they may have. The topics of each session are detailed in Table 2. Written educational materials will be provided to support the classes in both interventions.

**Table 2:** Overview of the Resilience, Stress, and Ethnicity (RiSE) intervention and health education program (HEP) topics.

Session	RiSE program (intervention)	HEP (active control)
1	Introduction to RiSE; establish group as safe place for dialogue about racism and oppression	Introduction to HEP
2	Explore issues related to being a woman from a minoritized community and impact of intersectionality; discuss insidious forms of racism	Caring for your skin cleansers and makeup
3	Explore challenges in having conversations about race and racism and discuss how defensiveness makes conversations unproductive	Cooking organically
4	Identify both overt and covert racism; reflect how racism can affect emotions and esteem	Finding credible health care information on the web
5	Identify aversive racism, institutional racism, and the role of White privilege; reflect on how these forms of racism can affect emotions and esteem	Immunizations for adults—what is available and what do you need?
6	Describe the use of cognitive behavioral theory to conceptualize emotional reactions to race-based stress	Over-the-counter medications: potential hazards
7	Review physical reactions to stress; practice strategies to cope with stress	Growing an herb garden
8	Review resilience, coping, and empowerment in response to race-based stress	Communicating with the health care team
Booster 1	Discuss challenges and successes in coping with chronic stressors; reinforce the use of adaptive coping skills	Review of health education topics, questions, and answers
Booster 2	Discuss challenges and successes; reinforce the use of adaptive coping skills	Review of health education topics, questions, and answers

#### RiSE Intervention

The RiSE intervention was adapted by Motley and Shawahin (Motley D, unpublished data, November 2017) for a behavioral health setting using components from an open-ended intervention developed by Carlson et al [35]. During our pilot work, the intervention was provided face-to-face and was tailored to help African American women address the unique challenges of navigating >1 marginalized identity (ie, intersectionality) [83]. RiSE includes three main components: (1) processing and sharing experiences associated with race-based stress, (2) psychoeducation related to the biopsychosocial impacts of racism, and (3) skill building and empowerment [48]. The intervention is feasible and well accepted among African American women [55]. In the first several sessions, RiSE provides participants with a safe place to share the emotional impact of race-based stress and to offer supportive listening among others in the group. Participants discuss their experiences as African American women, considering the ways in which the interaction between their racial and gender identities shapes their experiences. Facilitators promote discussion related to addressing racism and the unique experiences of Black women (eg, being stereotyped as angry and being expected to embody the “strong Black woman” archetype) [84-86] at interpersonal and structural levels. The facilitators share evidence of strategies to promote effective communication and internal emotional regulation regarding experiences of racism. Next, participants are provided with information on how racism can be manifested and are encouraged to use a common language with which to discuss their experiences. The facilitators provide psychoeducation on intersectionality, structural racism [87], overt racism [88], microaggressions [89], and internalized racism [90]. In addition, the facilitators assist participants in using cognitive behavioral strategies to understand thoughts, feelings, and actions associated with their experiences. Finally, the facilitators teach participants about stress, anxiety, and the “alarm” system [91] as they pertain to race-based stressors and other chronic stressors. Participants are encouraged to use adaptive coping strategies as well as community-based and cultural coping strategies. Throughout the program, participants engage in self-monitoring of the practice of skills discussed during the group sessions. Participants are sequentially introduced to the empowerment process steps [45] through journaling homework. Several mechanisms are key to the RiSE intervention in alleviating stress-related symptoms [48]. First, the group-based format allows participants to share their experiences with others and verbally process traumatic events [92]. Shame is believed to be a key barrier to overcoming race-based trauma [93]. Allowing participants to share their inner thoughts and concerns in a safe environment permits them to process their experiences and gain emotional validation from their peers. In addition, cognitive restructuring surrounding race-based trauma is used. This helps the participants reshape the maladaptive beliefs, such as self-blame, internalized stigma, and hopelessness, associated with their experiences. Participants learn that they do not cause racial and gender-based discrimination; rather, their experiences are related to systemic racism [35]. Participants are also encouraged to discuss their methods of coping with discrimination and are taught the difference between unhealthy and healthy coping strategies. The facilitators share approaches, such as mindfulness, cognitive restructuring, and advocacy, that participants can use to cope with their experiences [35]. Finally, participants are empowered to identify the meaning of their experiences and celebrate successes in using adaptive coping strategies. Classes are facilitated by 2 clinical psychologists trained in RiSE and who have >5 years of experience facilitating the RiSE program. In addition, they are experienced in leading and managing group dynamics and will develop ground rules with participants in the first session, including how to ensure that all participants can participate in the discussion.

#### HEP Intervention

Topics for the HEP (active control group) sessions were identified so that they would not confound the overall objectives of the RiSE program [94]. Experienced speakers (eg, dietitian, pharmacist, and aesthetician) will facilitate the HEP classes in their area of expertise. Classes will focus on wellness promotion with topics that will include cooking organically, immunizations, and skincare.

Importantly, the RiSE intervention will be delivered using standardized materials by the same 2 experienced RiSE interventionists for all cohorts [95]. Participants are assigned weekly homework and maintain logs of their practice time. Strategies to monitor participant understanding and application of treatment include a weekly review of participant self-monitoring logs that document weekly practice. Facilitators will assess the understanding of RiSE skills by asking questions, discussing material with participants, and monitoring skills development during in-class practice. Participants complete a brief post-RiSE evaluation form to provide feedback regarding the use of RiSE skills during appropriate life situations. We will assess and control for group cohesion using the 5-item Engaged Scale from the Group Climate Questionnaire–Short Form [96,97]. The fidelity of the HEP control intervention will follow the methods proposed by Stanton et al [98] and will be similar to that of the treatment group. Session content will be reviewed and approved by principal investigators. Standardized PowerPoint (Microsoft Corporation) presentations and class materials will be used. Multiple mechanisms will be used to ensure the integrity of the RiSE and control interventions. These mechanisms support the 5 dimensions of intervention integrity formulated by Dane and Schneider [99]: adherence (the extent to which program objectives are met), quality of delivery (interventionist effectiveness), exposure (dosage), participant responsiveness (engagement), and program differentiation. Adherence to program objectives will be monitored using multiple informants and methods to collect integrity data. Interventionists will complete a self-evaluation checklist after each weekly session identifying the extent to which session objectives were met. The study team will review notes for patterns or any areas that need to be addressed.

### Outcome Variables

The outcome variables for the trial include stress-related symptoms (current perceived stress, depressive symptoms, fatigue, and sleep disturbance) and inflammatory burden (as indicated by biomarkers such as IL-6, TNF-α, IL-1β, IFN-γ, and salivary CRP; Table 3). Each of these variables will be collected at 4 time points: T0, T1, T2, and T3.

**Table 3:** Study measures.

		Enrollment	T0^a^	T1^b^	T2^c^	T3^d^
**Eligibility**						
	Eligibility screening questions	✓				
	Brief Screen for Cognitive Impairment [71]	✓				
**Aim 1: psychosocial stressors**						
	Chronic stress: Adult Stress and Adversity Inventory [100]		✓	✓	✓	✓
	Perceived discrimination: Detroit Area Study Discrimination Scale [101]		✓	✓	✓	✓
	Perceived racism: Index of Race-Related Stress–Brief Version B [102]		✓	✓	✓	✓
	Subjective social status: MacArthur Scale of Subjective Social Status [103]		✓	✓	✓	✓
**Aim 1: response to chronic stress exposure**						
	Current perceived stress: Perceived Stress Scale [104] and Stress Overload Scale [105]		✓	✓	✓	✓
	Depressive symptoms: Beck Depression Inventory (Motley D, unpublished data, November 2017); depressive symptoms: Patient Health Questionnaire-9 [106]		✓	✓	✓	✓
	Anxiety: General Anxiety Disorder-7 [107]					
	Fatigue: PROMIS^e^ Fatigue Short Form 8a [108]		✓	✓	✓	✓
	Sleep disturbance: PROMIS Short Form v1.0 [109]		✓	✓	✓	✓
	Inflammatory burden: salivary CRP^f^, TNF-α^g^, IL-6^h^, IL-1β^i^, IFN-γ^j^, and CRP^k^ (Human High Sensitivity Cytokine B Premixed Magnetic Luminex Performance Assay [R&D Systems])		✓	✓	✓	✓
**Aim 2: Coping strategies**						
	Coping: Ways of Coping Questionnaire [110]		✓	✓	✓	✓
	Coping with discrimination: Coping with Discrimination Scale [111]		✓	✓	✓	✓
	Internalizing racism: Appropriated Racial Oppression Scale [112]		✓	✓	✓	✓
	Resistance and empowerment: Resistance and Empowerment Against Racism Scale [113]		✓	✓	✓	✓
**Covariates and descriptive measures**						
	Early life adversity: Childhood Trauma Questionnaire [114]		✓			
	Ethnic identity: Vancouver Index of Acculturation [115]		✓			
	Health behaviors, anthropometrics, and brief medical history: Block Brief 2000 Food Frequency Questionnaire [116]; National Institute of Nursing Research Social Determinants of Health Survey [117]; Self-Administered Comorbidity Questionnaire [118]; blood pressure, height and weight, body fat percentage, and body water percentage [119]; Alcohol, Smoking, and Substance Involvement Screening Test [120]; Life Events Checklist-7 [121]; and Race-Related Events Scale [122], health behaviors, anthropometrics, brief medical history, and demographics		✓	✓	✓	✓
	Intervention and active control group cohesion: 5-Item Engaged Scale from Group Climate Questionnaire [96]			✓	✓	✓

^a^T0: baseline.

^b^T1: midintervention.

^c^T2: intervention completion.

^d^T3: 6 months after intervention completion.

^e^PROMIS: Patient-Reported Outcomes Measurement Information System.

^f^CRP: C-reactive protein.

^g^TNF-α: tumor necrosis factor–alpha.

^h^IL-6: interleukin-6.

^i^IL-1β: interleukin-1beta.

^j^IFN-γ: interferon-gamma.

^k^CRP is not measured using the Luminex Performance High Sensitivity. Cytokine Magnetic Panel B MultiPlex but rather an Enzyme-Linked Immunosorbent Assay kit from R&D Systems.

#### Inflammatory Biomarker Procedures

Saliva samples will be collected to measure inflammatory biomarkers because this method is less invasive than blood draws. Salivary CRP and proinflammatory cytokines (eg, IL-6, TNF-α, IL-1β, and IFN-γ) are associated with stress-related symptoms [46,82]. We will follow recent best practice recommendations for collecting salivary cytokines [123], including the use of the passive drool method for sample collection and returning specimens overnight on dry ice between sites. Saliva samples will be kept frozen at −80 °C before batch analysis.

#### Exposure Variables

Measurements of chronic stress (Adult Stress and Adversity Inventory), perceived discrimination (Detroit Area Study Discrimination Scale), perceived racism (Index of Race-Related Stress–Brief Version B), and subjective social status (MacArthur Scale of Subjective Social Status; Table 3) will be collected and analyzed as exposure variables. Trained interviewers will collect these data using an audio computer-assisted self-interview system [72].

#### Covariates

Several variables may influence or moderate the relationship between the intervention group and chronic stressors, stress-related symptoms, and inflammatory burden and coping mechanisms. For this reason, data on age, education, income, employment, height and weight, blood pressure, health behaviors, and early life adversities, as well as a brief medical history, will be collected at baseline and considered as covariates.

### Analytic Plan

#### Data Quality

Before data analysis, we will ensure the accuracy and integrity of the data by regularly reviewing and cleaning the data from both sites using frequency distribution to identify data entry errors. In addition, 50% of the data will be manually checked for accuracy. If the coding or entry error rate is higher than 2% for any individual test or instrument, all data for that particular test or instrument will be manually checked. Monitoring logs will be maintained for the following: (1) patients screened for participation (basic demographic information and reason for nonparticipation [for nonparticipants only]), (2) patient entry into the study, (3) timeliness of data collection, and (4) missing data. Regarding missing items from the psychometric measurements, each tool will be assessed for missing data, and, if appropriate, participants will be contacted to complete the missing items.

#### Statistical Analysis

Baseline demographics and clinical characteristics will be reported according to the CONSORT (Consolidated Standards of Reporting Trials) guidelines. The size of any chance group imbalances will be described, but no formal significance testing of group differences will be performed [124]. Generalized linear mixed models (GLMMs) will be used for analyses of outcomes due to their flexibility regarding the distribution of dependent variables (DVs) and their superiority over alternative approaches such as repeated measures ANOVA for longitudinal modeling with missing data. Data will be collected from participants at 4 time points (T0: baseline, T1: midintervention; 4 wk, T2: intervention completion; 8 wk, and T3: 6 mo after intervention completion). Time will be modeled as a fixed effect, and random intercepts and random slopes will be considered to account for within-individual differences and trends. The primary DVs include stress-related symptoms (current perceived stress, depressive symptoms, fatigue, and sleep disturbance) and biomarkers (ie, IL-6, TNF-α, IL-1β, IFN-γ, and CRP) to be modeled using GLMM. Before modeling, DVs will be plotted to choose an optimal distribution and link function for GLMMs and to identify potential floor or ceiling effects. For each DV, a GLMM will regress the follow-up DV on a group×time interaction, the baseline value of the DV, and covariates. Treatment assignment (group) will be a fixed effect, and adjusted means with SEs will be used to describe group differences. Random effects will be evaluated for site and cohort. Exploratory end points include coping (Ways of Coping Questionnaire and Coping with Discrimination Scale), internalizing racism (Appropriated Racial Oppression Scale), and resistance to racism (Resistance and Empowerment Against Racism), which will be DVs in separate GLMMs, as described earlier.

Covariate adjustment will be performed to account for explained variation in outcome variables [125]. Not all covariates considered may be necessary to include in the models to explain the bulk of the variation in DVs; therefore, a parsimonious subset will be chosen concerning multicollinearity, the strength of associations, and appropriateness for total sample size. Residual plots will be examined to determine whether transformations or other regression remedial measures are required to satisfy model assumptions. Although sample size calculations have accounted for potential attrition, analyses of end points will be conducted under the intent-to-treat principle. While every attempt will be made to capture all available data on participants, we may consider sensitivity analyses using model-based approaches (eg, multiple imputation) for missing data [126]. A detailed statistical analysis plan will be prepared and finalized by the study biostatistician before the database lock to promote the reproducibility and transparency of the analytic approach. Analyses will be performed using SAS (version 9.4; SAS Institute) and R (version 4.0.2 or later; R Foundation for Statistical Computing). Results reporting will include all preplanned analyses, and any post hoc analyses will be clearly labeled as such when we disseminate this work.

### Sample Size

We will enroll 300 participants and anticipate that at least 250 (n=125, 50% per arm) will have the 6-month postintervention follow-up data. For the primary end point of the Perceived Stress Scale (PSS), this sample size has >80% power to detect a 3-point difference in mean PSS scores at follow-up using an *F* test in an analysis of covariance. This assumes the R-squared value of 5 covariates, including baseline PSS, to be 0.3 and uses a significance level of α=.05. This calculation conservatively estimates the SD of PSS to be 10 points. Initial psychometric studies and norm groups analysis of the PSS reported SDs of 7.2 points in African Americans; relevant to the RiSE study, group SDs ranged from 6 to 10 points from a pilot trial involving African Americans with hypertension [127]. At this sample size and with similar design assumptions, effect sizes were estimated using the RiSE pilot study for the hypothesized mediator of coping as well as biomarkers [48]. The estimates from the analysis of covariance (Table 4) represent the lower limits for power because the mixed model approach that incorporates all time points may be more powerful in detecting meaningful differences.

**Table 4:** Pilot study estimates and effect sizes.

Outcome evaluated at 6 months	RiSE^a^ group, mean	Control group, mean	|Δ|, mean	Pooled SD	Observed effect size	Mean difference required for 80% power^b^
WCQ^c^: active coping	23.76	24.77	1.01	8.24	0.06	2.45
WCQ: avoidance coping	8.14	14.38	6.24	5.25	0.59	1.56
WCQ: minimize situation	11.21	14.04	2.83	4.36	0.32	1.30
TNF-α^d^	0.95	1.00	0.05	0.36	0.07	0.11
hsCRP^e^	3.83	7.24	3.41	6.30	0.27	1.88

^a^RiSE: Resilience, Stress, and Ethnicity.

^b^Assumes n=125 per group and α=.05; R-squared value of 5 covariates is 0.30.

^c^WCQ: Ways of Coping Questionnaire.

^d^TNF-α: tumor necrosis factor–alpha.

^e^hsCRP: high-sensitivity C-reactive protein.

## Results

**Figure 3:**
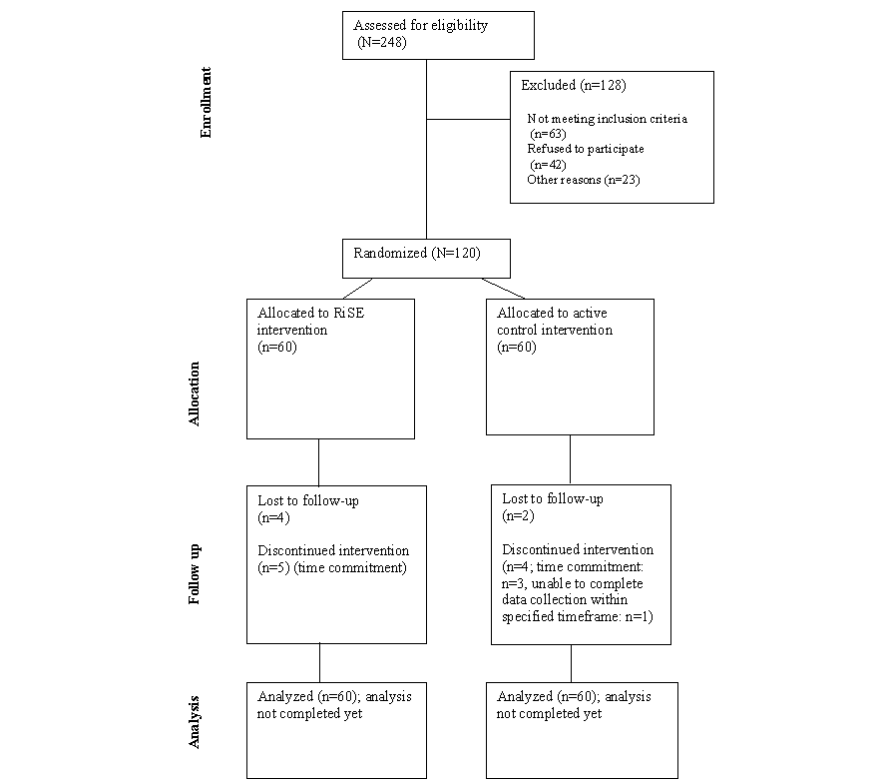
CONSORT (Consolidated Standards of Reporting Trials) flow diagram for the study. RiSE: Resilience, Stress, and Ethnicity.

This study was funded by the National Institute on Aging on March 30, 2023, with a project end date of January 31, 2028. Recruitment and enrollment began in October 2023 and is ongoing. As of March 2025, we enrolled 120 participants. Figure 3 depicts the CONSORT flow diagram for study participants as of March 2025.

Table 5 presents the start dates and enrollment for each cohort. We plan to enroll 3 cohorts each year with anticipated enrollment completed in 2027. In addition, retention (defined as attending at least 5 of the 8 program classes) has been 95% (38/40) for the RiSE intervention and 91% (41/45) for the HEP active control intervention. We began preliminary analysis of baseline data in March 2025.

**Table 5:** Cohort start dates and enrollment size (n=120).

Cohort	Start date	Participants, n (%)
1	October 30, 2023	23 (19.2)
2	February 26, 2024	23 (19.2)
3	May 7, 2024	26 (21.7)
4	September 16, 2024	26 (21.7)
5	February 17, 2025	22 (18.3)

## Discussion

### Principal Findings

This study proposes to examine the effect of a race-based stress reduction intervention, called RiSE, on stress-related symptoms, coping, and inflammation in older African American women at risk for CMD. We anticipate that participants completing the RiSE program will have decreased stress-related symptoms, improved adaptive coping, and decreased inflammatory burden compared to those randomized to the active control group (HEP).

Racism is dangerous, pervasive, and unfortunately a growing problem in the United States. A third of African Americans report experiencing a race-related incident in the past year [128]. Undeniably, racism needs to be addressed at the interpersonal, community, and societal levels. However, until such time, ways to reduce the impact of racism on psychological and physical health at the individual level must be developed and tested. The RiSE intervention may accomplish this goal. It reduces stress and has the potential to reduce the psychobiological consequences of racism and discrimination, leading to decreased inflammation and ultimately contributing to lowered CMD and CVD risk in African American women. In sum, despite evidence that experiencing racism and perceived discrimination heightens the inflammatory response and CMD [19,53], few evidence-based interventions are available to help individuals from minoritized communities cope with unique stressors related to their racial or ethnic identity. There is evidence that health behaviors [129], early life adversity [130,131], and ethnic identity [132-134] may impact the relationships among chronic stressors (perceived discrimination, racism, and subjective social status) and the psychological and inflammatory response to stress.

### Limitations and Strengths

The proposed study has some limitations that should be recognized. As with most RCTs, challenges related to recruitment and retention are anticipated. In response, we have developed a detailed recruitment plan supported by our community advisory board as well as strategies to minimize attrition, including immediately contacting participants who miss program classes or data collection visits as well as providing fair compensation. In addition, generalizability is a potential limitation of this study. Future work will examine RiSE in additional populations. Despite these challenges, this study will be one of the first to examine a race-based stress reduction intervention in African American women and has the potential to improve the health of minoritized groups faced with the chronic stress associated with experiencing racism and discrimination [35,39-45,48,55] (Motley D, unpublished data, November 2017). In addition, RiSE is being offered via Zoom, which may allow participants who cannot travel or have caregiving responsibilities the opportunity to participate in the program. If RiSE is found to be effective in older African American women, future research may consider evaluating RiSE in other populations, such as younger African American women as well as men.

## Abbreviations

CBT: cognitive behavioral therapy
CMD: cardiometabolic disease
CONSORT: Consolidated Standards of Reporting Trials
CRP: C-reactive protein
CVD: cardiovascular disease
DV: dependent variable
GLMM: generalized linear mixed model
HEP: health education program
IFN-γ: interferon-gamma
IL-1β: interleukin-1beta
IL-6: interleukin-6
MBSR: mindfulness-based stress reduction
PSS: Perceived Stress Scale
RCT: randomized controlled trial
REDCap: Research Electronic Data Capture
RiSE: Resilience, Stress, and Ethnicity
TNF-α: tumor necrosis factor–alpha

## Data Availability

This study is in the data collection phase. Datasets will be made available at a later time upon request from the corresponding author.
